# Probiotic supplementation modulates the gut microbiome and improves clinical outcomes in pediatric refractory asthma

**DOI:** 10.3389/fmicb.2026.1756436

**Published:** 2026-02-11

**Authors:** Zheng Liu, Wen Deng, Wenlin Xu, Linlin Ye, Zhihui Rao

**Affiliations:** 1 Children’s Hospital of Chongqing Medical University, National Clinical Research Center for Children and Adolescents Health and Diseases, Ministry of Education, Key Laboratory of Child Development and Disorders, Chongqing Key Laboratory of Pediatric Metabolism and Inflammatory Diseases, Chongqing, China; 2 Department of Pediatric, Jiangxi Children’s Medical Center, Nanchang, Jiangxi, China

**Keywords:** gut microbiome, gut-lung axis, pediatric, probiotics, refractory asthma

## Abstract

**Background:**

Refractory asthma in children remains a clinical challenge despite conventional therapies, with emerging evidence linking gut microbiome dysbiosis to persistent inflammation via the gut-lung axis. This study investigated whether multi-strain probiotic supplementation could improve asthma control and restore microbial balance when added to standard treatment.

**Methods:**

This prospective randomized controlled trial enrolled 88 children aged 4–8 years with refractory asthma. Participants were allocated into two groups (*n* = 44 each): a conventional treatment group (bronchodilators and glucocorticoids) and a combination treatment group, which received conventional therapy plus a multi-strain probiotic (*Bifidobacterium*, *Lactobacillus acidophilus*, *Streptococcus thermophilus*) for 4 months. Primary outcomes were asthma control level, Asthma Control Test (ACT) scores, and pulmonary function (FEV₁, FVC, PEF). Secondary outcomes included gut microbiota changes, assessed by 16S rRNA gene sequencing.

**Results:**

Combination therapy achieved complete asthma control in 68.18% of patients versus 36.36% with conventional therapy (*Z* = 2.415, *p* < 0.05). Post-treatment ACT scores were higher in the combination group (22.45 ± 1.20 vs. 19.78 ± 1.45; *p* < 0.05), with superior improvements in FEV1 (2.65 ± 0.10 L vs. 2.30 ± 0.08 L; *p* < 0.001), FVC (3.10 ± 0.18 L vs. 2.80 ± 0.15 L; *p* < 0.001), and PEF (4.00 ± 0.25 L/s vs. 3.50 ± 0.20 L/s; p < 0.001). Symptoms resolved faster with combination therapy (e.g., cough: 5.60 ± 1.50 vs. 10.45 ± 2.30 days; *p* < 0.05). Microbiome analysis showed increased alpha diversity (e.g., Shannon index: *p* < 0.05) and beneficial shifts in the combination group, including higher Bifidobacterium (25.00 ± 15.31% vs. 0.98 ± 1.92%; *p* < 0.001) and reduced Bacteroides, with distinct beta diversity clustering (PERMANOVA *p* < 0.05).

**Conclusion:**

Adjunctive multi-strain probiotics enhance clinical outcomes and gut microbiome health in pediatric refractory asthma, supporting microbiome-targeted therapies via the gut-lung axis. Larger, double-blind randomized controlled trials are warranted to confirm long-term benefits.

## 1 Introduction

Childhood asthma affects over 300 million individuals globally and represents one of the most prevalent chronic respiratory disorders in pediatrics (Eapen et al., 2025). Refractory asthma, characterized by persistent symptoms despite optimal conventional therapy, poses particular challenges for clinical management (Wedayanti, 2023). While targeted biological therapies have emerged, a substantial proportion of pediatric patients continue experiencing inadequate disease control, highlighting the need for innovative therapeutic approaches.

The gut-lung axis has emerged as a critical pathway in respiratory disease pathogenesis, with bidirectional communication between the gastrointestinal tract and pulmonary system (Ciprandi et al., 2023). Gut microbiota modulate lung immunity through multiple mechanisms, including short-chain fatty acid production, regulation of inflammatory mediators, and immune cell trafficking (Logoń et al., 2023). The “microbiota hypothesis” suggests that early-life bacterial exposures shape microbiota composition and influence subsequent susceptibility to allergic diseases, including asthma (Jiang et al., 2025).

Children with asthma frequently exhibit dysbiosis in both gut and lung microbiomes, characterized by reduced microbial diversity and altered bacterial composition (Indolfi et al., 2023; Kim et al., 2024; Kesavelu and Jog, 2023). This dysbiosis typically features decreased beneficial bacteria (Bifidobacterium, Lactobacillus) and increased potentially inflammatory taxa (certain Bacteroides and Clostridium species) (Cooper et al., 2023; Sampaio Dotto Fiuza et al., 2024). Such microbial imbalance disrupts immune homeostasis, perpetuating the chronic inflammation characteristic of asthma (Wang et al., 2018).

Probiotic supplementation has emerged as a potential therapeutic strategy to restore microbial balance and modulate immune responses (Merenstein et al., 2024). Meta-analyses suggest that probiotics during critical developmental windows can reduce allergic disease risk, with strain-specific and dose-dependent effects (Zhou et al., 2023). Specific probiotic strains can modulate dendritic cell maturation, enhance regulatory T-cell function, and promote anti-inflammatory metabolite production (Kesavelu and Jog, 2023).

However, the efficacy of probiotics in managing established pediatric refractory asthma remains inadequately characterized. Most previous studies have been small-scale, focused primarily on clinical outcomes without comprehensive microbiome assessment (Merenstein et al., 2024; Waitzberg et al., 2024). The optimal bacterial strains, dosing, and patient selection criteria remain undefined. Multi-strain formulations targeting multiple immune pathways may offer advantages over single-strain interventions (Zhou et al., 2023; Zhang et al., 2018; Bannier et al., 2019; McFarland, 2021).

This prospective randomized controlled trial evaluates the clinical efficacy of multi-strain probiotic supplementation combined with conventional therapy in children with refractory asthma. Through assessment of asthma control, pulmonary function, and gut microbiome alterations via 16S rRNA sequencing, we aimed to establish the therapeutic potential of microbiome-targeted interventions and elucidate underlying mechanisms.

## 2 Materials and methods

### 2.1 Study design and participants

This prospective study was conducted at the Respiratory Department of Jiangxi Children’s Medical Center from June 2023 to May 2024. A total of 120 children diagnosed with refractory asthma were consecutively enrolled based on their order of presentation, which served as unique identifiers for randomization. Participants were randomly divided into two groups: the conventional treatment group (*n* = 60) and the combination treatment group (*n* = 60). Randomization was performed using a simple allocation method based on the enrollment sequence to ensure balanced distribution. The study was open-label, with no blinding of participants, investigators, or outcome assessors, as the interventions involved distinct therapeutic additions that precluded masking.

Following enrollment, 32 children were excluded (16 per group): 8 per group due to inadequate intestinal microbiota test results, 5 per group for non-adherence to probiotic administration (combination group only, but balanced reporting), and 3 per group for incomplete conventional treatment course. Thus, 88 children were included in the final analysis, with 44 in each group. Baseline characteristics, including age, gender, body mass index (BMI), disease duration, allergic history, and comorbid allergic conditions (such as rhinitis and eczema), were comparable between groups.

Inclusion Criteria

- Diagnosis of childhood refractory asthma according to established guidelines (McFarland, 2021).
- Age between 4.0 and 8.0 years.
- Residence in local or surrounding areas.
- Signed informed consent from parents or legal guardians.
- Ability of children and families to cooperate with examinations and questionnaires.

Exclusion Criteria

- Severe digestive system diseases (e.g., severe diarrhea, vomiting, or gastrointestinal infection) within the last 30 days.
- Comorbidities such as congenital heart disease, congenital respiratory tract malformations, or primary immunodeficiency diseases.
- Use of probiotics within the last 30 days or antibiotics for more than 5 days in the past 30 days.
- Receipt of other immunomodulators or biological agents in the past 3 months.
- Poor compliance with treatment protocols.

The study protocol was approved by the Medical Ethics Committee of Jiangxi Children’s Medical Center (approval number ADC-20242066). All procedures adhered to ethical standards, including the Declaration of Helsinki. Informed consent included detailed information on potential risks, benefits, and the right to withdraw at any time without prejudice.

### 2.2 Interventions

Participants in the conventional treatment group received standard therapy for refractory asthma, including bronchodilators (e.g., salbutamol aerosol) and glucocorticoids (e.g., budesonide aerosol suspension) administered via nebulization. Doses and frequencies were tailored to the child’s age and disease severity in accordance with relevant diagnostic and treatment guidelines.

The combination treatment group received the same conventional therapy plus probiotic-based intestinal microbiota balance therapy. The probiotic formulation (Beijing Kang Yisheng Biotechnology Co., Ltd.; implementation standard GB/T 29602-2013; production license SC20131011400336) contained three strains: *Bifidobacterium longum* BL999, *Lactobacillus acidophilus* La-14, and *Streptococcus thermophilus* ST-21, with a total of approximately 1 × 10^9^ CFU viable bacteria per bag. One bag was administered twice daily (morning and evening), dissolved in warm water below 37 °C. Sachets were stored at room temperature (<25 °C) in a dry place. Adherence was monitored through family self-report, monthly returned sachet counts during clinic/telephone follow-up, and direct inquiry about administration. Administration involved one bag (containing 1 × 10^9^ CFU total viable bacteria per bag) twice daily (morning and evening), dissolved in warm water below 37 °C. Interventions lasted 4 months for both groups.

Elimination criteria during the study included severe adverse reactions (e.g., severe diarrhea, vomiting, or allergic responses), disease deterioration, or exacerbation attributable to study drugs. Follow-up assessments were conducted monthly via clinic visits or telephone to monitor adherence, adverse events, and treatment progress. An Excel-based form was used to record patient demographics, intervention details, and outcomes.

### 2.3 Outcome measures

Primary outcomes focused on asthma control and clinical efficacy. Asthma control levels were evaluated per the Guidelines for Diagnosis and Prevention of Bronchial Asthma in Children, integrating daytime and nighttime symptoms, activity limitations, and emergency drug use, categorized as complete control, partial control, or uncontrolled.

The parent/guardian-proxy version of the Childhood Asthma Control Test (C-ACT), a 5-item questionnaire with a maximum of 25 points, assessed symptom control; higher scores indicated better control. Lung function was measured using a pulmonary function detector, reporting absolute values for forced expiratory volume in the first second (FEV₁, L), forced vital capacity (FVC, L), and peak expiratory flow (PEF, L/s). Percent-predicted values were calculated internally against local age-, height-, and sex-adjusted pediatric reference norms but are not reported here. Measurements were taken before and after the 4-month intervention.

Symptom resolution times for cough, shortness of breath, expectoration, and wheezing rales were recorded to evaluate treatment rapidity.

Secondary outcomes involved gut microbiota analysis. Stool samples were collected pre- and post-intervention for 16S rRNA gene sequencing, performed by Shanghai Jing Yibai Biotechnology Co., Ltd. Total DNA was extracted, and the V3-V4 region was amplified for sequencing to assess microbial abundance and diversity.

### 2.4 Sample collection and processing

Stool specimens were collected by families, focusing on the middle portion to avoid contamination with urine or other fluids, and stored in designated tubes before shipment for analysis.

DNA extraction used the FastDNA® Spin Kit for Soil (MP Biomedicals, USA). Steps included adding 0.5 g stool sample, 978 μL Sodium Phosphate Buffer, and 122 μL MT Buffer to a Lysing Matrix E tube; homogenization at 6 m/s for 40 s; centrifugation at 14,000 rpm for 10 min; supernatant transfer and addition of 250 μL PPS; further centrifugation; binding with 900 μL matrix; washing with 500 μL 5.5 M guanidine isothiocyanate; and elution to yield total DNA.

DNA quality was assessed via NanoDrop2000 for concentration and purity, and 1% agarose gel electrophoresis for integrity.

PCR amplification targeted the 16S V3-V4 region using primers 338F (ACTCCTACGGGAGGCAGCAG) and 806R (GGACTACHV GGGTWTCTAAT). The reaction system comprised 4 μL 5 × FastPfu Buffer, 2 μL 2.5 mM dNTPs, 0.8 μL each primer (5 μM), 0.4 μL FastPfu Polymerase, 0.2 μL BSA, 10 ng template DNA, and ddH₂O to 20 μL. Cycling parameters were: 95 °C for 3 min; 27 cycles of 95 °C for 30 s, 55 °C for 30 s, 72 °C for 45 s; and 72 °C for 10 min.

Key reagents included agarose (Biowest, Spain), FastPfu Polymerase (TransGen, China), AxyPrep DNA Gel Extraction Kit (Axygen, USA), NEXTFLEX® Rapid DNA-Seq Kit (Bioo Scientific, USA), and MiSeq Reagent Kit v3 (Illumina, USA). Instruments encompassed Eppendorf pipettes, centrifuges (Aikomai, China; Hitachi, Japan), Vortex-Genie 2 mixer (Scientific Industries, USA), FastPrep-24 5G bead beater (MP, USA), Quantus™ Fluorometer (Promega, USA), DYCP-31DN electrophoresis apparatus (Beijing June 1, China), ABI GeneAmp® 9,700 PCR system (ABI, USA), Illumina MiSeq sequencer (Illumina, USA), and BioTek ELx800 microplate reader (Biotek, USA).

### 2.5 Microbiota analysis

Alpha diversity, reflecting within-sample species richness and evenness, was evaluated using Chao1, observed species, Shannon, and Simpson indices. Differences were compared via Wilcoxon test.

Beta diversity, assessing between-sample differences, was calculated using UniFrac distances (weighted and unweighted). Principal coordinate analysis (PCoA) and phylogenetic tree clustering visualized results, with PERMANOVA testing significance.

### 2.6 Statistical analysis

Data were analyzed using SPSS 26.0 (IBM, USA). Continuous variables are presented as mean ± standard deviation, with intergroup differences assessed by independent-samples *t*-test. Categorical data are expressed as frequencies (%), compared via *χ*^2^ test. Ordinal data (asthma control levels) were analyzed using Wilcoxon rank-sum test. *p* < 0.05 indicated statistical significance. No adjustments for multiple comparisons were applied, as the analyses were exploratory and aligned with the study’s objectives.

## 3 Results

### 3.1 Participant characteristics and flow

**Figure 1:**
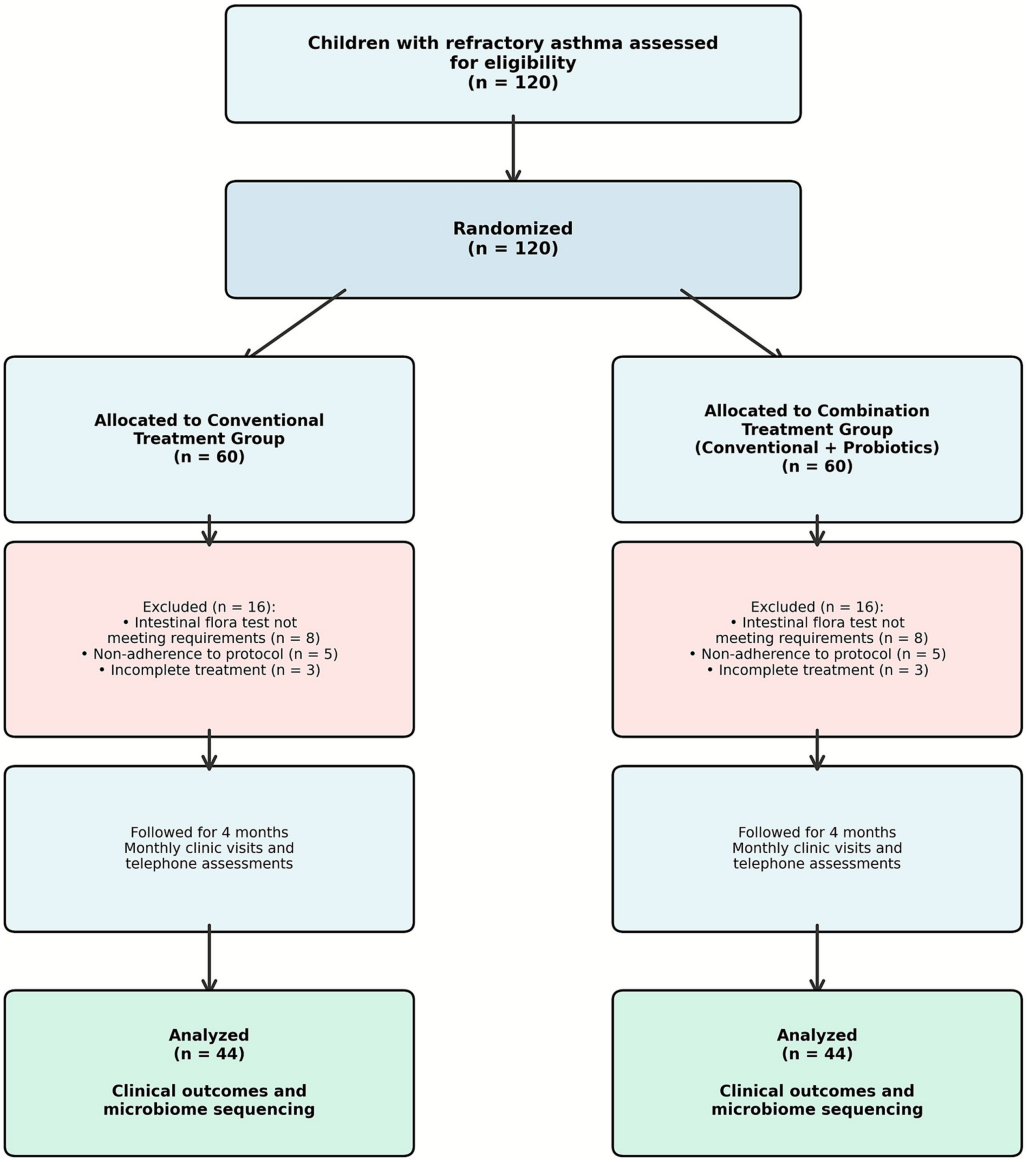
Participant flow diagram. Flow diagram illustrating participant enrollment, randomization, follow-up, and analysis in a prospective randomized controlled trial of probiotic supplementation for pediatric refractory asthma. A total of 120 children with refractory asthma were assessed for eligibility and randomized to either the conventional treatment group (*n* = 60) or the combination treatment group receiving conventional therapy plus probiotics (*n* = 60). A total of 32 participants were excluded (16 per group): eight per group due to inadequate intestinal microbiota test results, five per group for non-adherence to the probiotic protocol, and three per group for incomplete conventional treatment. Monthly follow-up assessments were conducted via clinic visits and telephone monitoring. Final analysis included 44 participants per group (total *n* = 88).

Between June 2023 and May 2024, 60 children with refractory asthma were enrolled and allocated to study groups at the Respiratory Department of Jiangxi Children’s Medical Center. Following balanced exclusions across groups (16 per group: 8 per group for inadequate intestinal microbiota test results, 5 per group for non-adherence to probiotic protocol, and 3 per group for incomplete conventional treatment), 88 participants (44 per group) completed the study protocol (Figure 1).

Baseline demographic and clinical characteristics demonstrated comparable distributions between groups. The conventional treatment group comprised 26 males and 18 females with mean age 5.42 ± 0.85 years, while the combination treatment group included 24 males and 20 females aged 5.38 ± 0.79 years (*p* = 0.857). Body mass index was similar between groups (17.56 ± 1.20 vs. 17.48 ± 1.15 kg/m^2^, *p* = 0.812), as was disease duration (3.12 ± 1.10 vs. 3.08 ± 1.05 months, *p* = 0.899). Comorbid allergic conditions showed no significant between-group differences, with rhinitis present in 16/44 (36.4%) conventional treatment participants versus 18/44 (40.9%) combination treatment participants (*p* = 0.752), and eczema history documented in 24/44 (54.5%) versus 22/44 (50.0%) respectively (*p* = 0.764) (Table 1). Exclusions were balanced across groups (e.g., 8 per group for inadequate microbiota test results), with no significant differences in baseline characteristics post-exclusion, minimizing potential bias.

**Table 1:** Baseline characteristics of study participants.

Characteristic	Conventional treatment (*n* = 44)	Combination treatment (*n* = 44)	*p*-value
Age (years), mean ± SD	5.42 ± 0.85	5.38 ± 0.79	0.857
Sex, *n* (%)			0.731
Male	26 (59.1)	24 (54.5)	
Female	18 (40.9)	20 (45.5)	
BMI (kg/m^2^), mean ± SD	17.56 ± 1.20	17.48 ± 1.15	0.812
Disease duration (months), mean ± SD	3.12 ± 1.10	3.08 ± 1.05	0.899
Comorbid conditions, *n* (%)			
Allergic rhinitis	16 (36.4)	18 (40.9)	0.752
Eczema history	24 (54.5)	22 (50.0)	0.764

SD, standard deviation; BMI, body mass index. *p*-values from independent *t*-test for continuous variables and chi-square test for categorical variables.

### 3.2 Primary outcome: asthma control level

After 4 months of treatment, the combination therapy group demonstrated markedly superior asthma control compared to conventional treatment alone. Complete asthma control was achieved in 30/44 (68.18%) children receiving combination therapy versus 16/44 (36.36%) in the conventional treatment group. Partial control was documented in 12/44 (27.27%) combination therapy participants compared to 18/44 (40.91%) conventional treatment participants, while uncontrolled asthma persisted in only 2/44 (4.55%) combination therapy participants versus 10/44 (22.73%) conventional treatment participants. This distribution of control levels showed statistically significant between-group differences (*Z* = 2.415, *p* < 0.05), indicating superior overall asthma control with probiotic supplementation (Table 2). The *Z*-value (*Z* = 2.415, *p* < 0.05) was derived from the Wilcoxon rank-sum test, appropriate for comparing ordinal asthma control levels between groups.

**Table 2:** Asthma control levels after 4-month treatment.

Control level	Conventional treatment *n* (%)	Combination treatment *n* (%)
Complete control	16 (36.4)	30 (68.2)
Partial control	18 (40.9)	12 (27.3)
Uncontrolled	10 (22.7)	2 (4.5)

Wilcoxon rank-sum test (Mann–Whitney U test): *Z* = 2.415, *p* < 0.05.

*p < 0.05; **p < 0.01; ***p < 0.001 indicate statistical significance.

### 3.3 Asthma control test scores

Baseline ACT scores were comparable between groups (16.92 ± 1.08 vs. 16.85 ± 1.12, *p* > 0.05). Following 4 months of treatment, both groups demonstrated improvement, however, the magnitude of improvement differed substantially. The combination treatment group achieved mean post-treatment ACT scores of 22.45 ± 1.20, representing a significant increase from baseline (*t* = 5.678, *p* < 0.05). The conventional treatment group reached post-treatment scores of 19.78 ± 1.45, showing modest improvement from baseline (*t* = 1.234, *p* > 0.05). The between-group difference in post-treatment ACT scores was statistically significant, favoring combination therapy (Table 3).

**Table 3:** Asthma control test (ACT) scores.

Treatment group	Baseline score	Post-treatment score	Within-group *p*-value	Between-group *p*-value†
Conventional treatment	16.85 ± 1.12	19.78 ± 1.45	0.134	
Combination treatment	16.92 ± 1.08	22.45 ± 1.20	<0.001***	<0.001***

Values are presented as mean ± SD. Within-group comparisons were performed using paired *t*-tests.

†Between-group comparisons of post-treatment scores were performed using independent-samples *t*-tests.

*p < 0.05; **p < 0.01; ***p < 0.001 indicate statistical significance.

### 3.4 Pulmonary function parameters

Baseline pulmonary function measurements showed no significant between-group differences for FEV1 (1.30 ± 0.03 L vs. 1.28 ± 0.04 L), FVC (2.52 ± 0.10 L vs. 2.54 ± 0.12 L), or PEF (3.18 ± 0.12 L/s vs. 3.20 ± 0.15 L/s) (all *p* > 0.05). Post-treatment assessments revealed substantial improvements in the combination treatment group across all parameters. FEV1 increased to 2.65 ± 0.10 L in the combination group (*t* = 10.456, *p* < 0.001) versus 2.30 ± 0.08 L in the conventional group (*t* = 1.532, *p* = 0.134). FVC reached 3.10 ± 0.18 L with combination treatment (*t* = 8.765, *p* < 0.001) compared to 2.80 ± 0.15 L with conventional treatment alone (*t* = 1.234, *p* = 0.228). PEF improved to 4.00 ± 0.25 L/s in combination therapy recipients (*t* = 9.876, *p* < 0.001) versus 3.50 ± 0.20 L/s in conventional treatment recipients (*t* = 1.567, *p* = 0.125) (Table 4).

**Table 4:** Pulmonary function parameters before and after treatment.

Parameter	Treatment group	Baseline	Post-treatment	Change from baseline	*p*-value*
FEV₁ (L)	Conventional	1.28 ± 0.04	2.30 ± 0.08	+1.02 (79.7%)	0.134
	Combination	1.30 ± 0.03	2.65 ± 0.10	+1.35 (103.8%)	<0.001***
FVC (L)	Conventional	2.54 ± 0.12	2.80 ± 0.15	+0.26 (10.2%)	0.228
	Combination	2.52 ± 0.10	3.10 ± 0.18	+0.58 (23.0%)	<0.001***
PEF (L/s)	Conventional	3.20 ± 0.15	3.50 ± 0.20	+0.30 (9.4%)	0.125
	Combination	3.18 ± 0.12	4.00 ± 0.25	+0.82 (25.8%)	<0.001***

Values are presented as mean ± SD. FEV₁, forced expiratory volume in 1 s; FVC, forced vital capacity; PEF, peak expiratory flow. *p-values from paired *t*-tests for within-group comparisons.

*p < 0.05; **p < 0.01; ***p < 0.001 indicate statistical significance.

Values represent absolute volumes and flow rates (not percent-predicted).

Although absolute improvements in pulmonary function were observed in both groups (reflecting the effect of continued conventional therapy), only the combination group achieved statistical significance due to the greater magnitude and consistency of responses.

### 3.5 Time to symptom resolution

The combination treatment group experienced significantly accelerated resolution of respiratory symptoms compared to conventional treatment. Cough resolved within 5.60 ± 1.50 days with combination therapy versus 10.45 ± 2.30 days with conventional treatment (*t* = 7.123, *p* < 0.05). Dyspnea improved within 3.05 ± 1.02 days in the combination group compared to 5.32 ± 1.50 days in the conventional group. Expectoration ceased after 3.70 ± 0.85 days with combination treatment versus 5.48 ± 1.60 days with conventional treatment alone. Auscultatory wheezing disappeared within 2.55 ± 0.70 days in combination therapy recipients compared to 3.92 ± 1.20 days in conventional treatment recipients (*t* = 5.234, *p* < 0.05) (Table 5).

**Table 5:** Time to resolution of clinical symptoms (days).

Symptom	Conventional treatment	Combination treatment	*p*-value
Cough	10.45 ± 2.30	5.60 ± 1.50	<0.001***
Dyspnea	5.32 ± 1.50	3.05 ± 1.02	<0.001***
Expectoration	5.48 ± 1.60	3.70 ± 0.85	<0.001***
Wheezing on auscultation	3.92 ± 1.20	2.55 ± 0.70	<0.001***

Values are mean ± SD days. *p*-values from independent *t*-test.

*p < 0.05; **p < 0.01; ***p < 0.001 indicate statistical significance.

### 3.6 Sequencing quality and coverage

**Figure 2:**
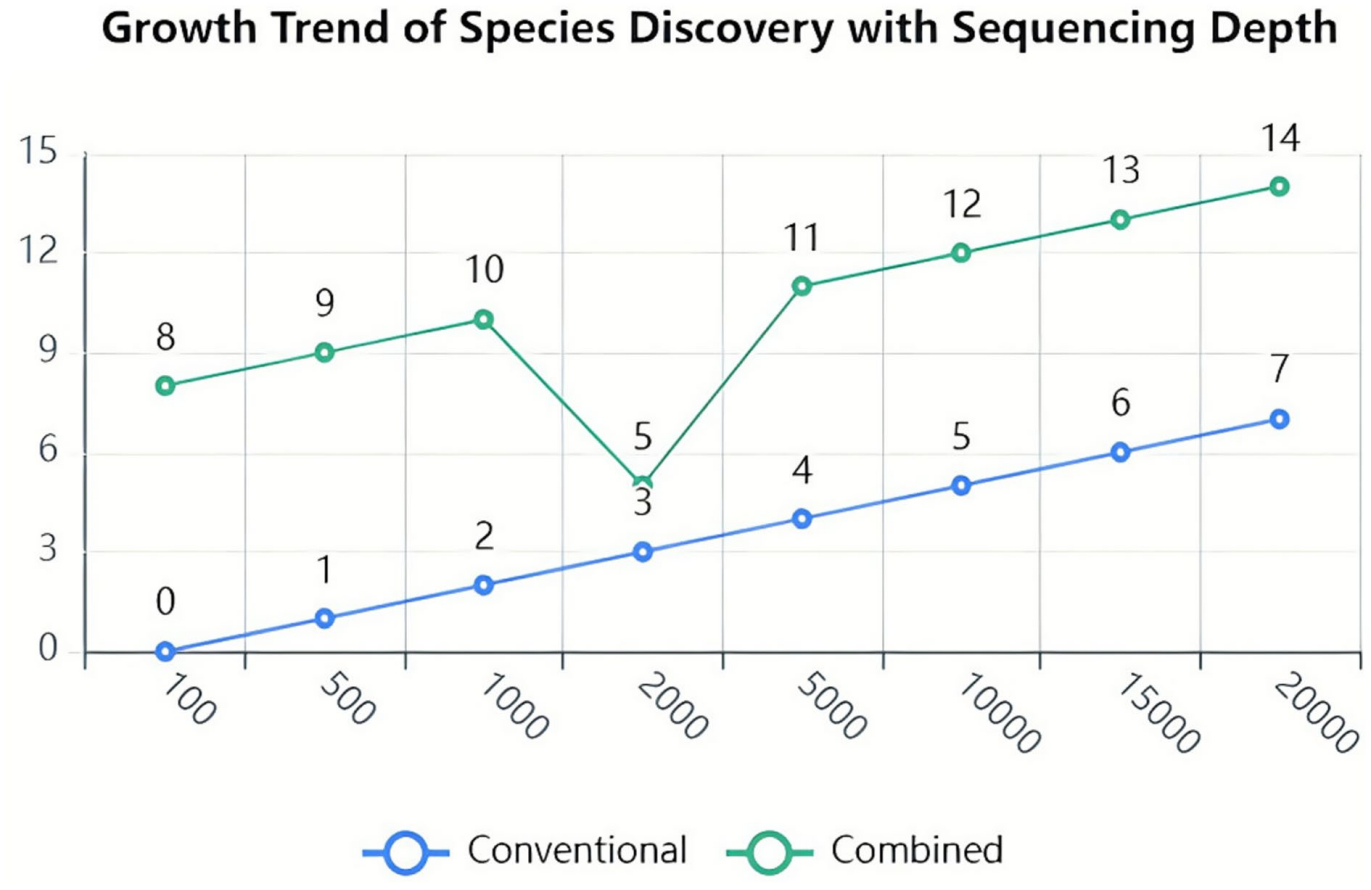
Rarefaction curves of the stool samples. Rarefaction curves showing the relationship between sequencing depth and species richness across all fecal samples. The *x*-axis represents the number of sequences sampled, while the y-axis represents the number of observed operational taxonomic units (OTUs). The curves plateau at approximately 1,000 sequences, indicating that the sequencing depth (>15,000 sequences per sample) was sufficient to capture the majority of microbial diversity present in the samples. Each line represents an individual sample from the conventional treatment group (before and after treatment) and the combination treatment group (before and after treatment).

**Figure 3:**
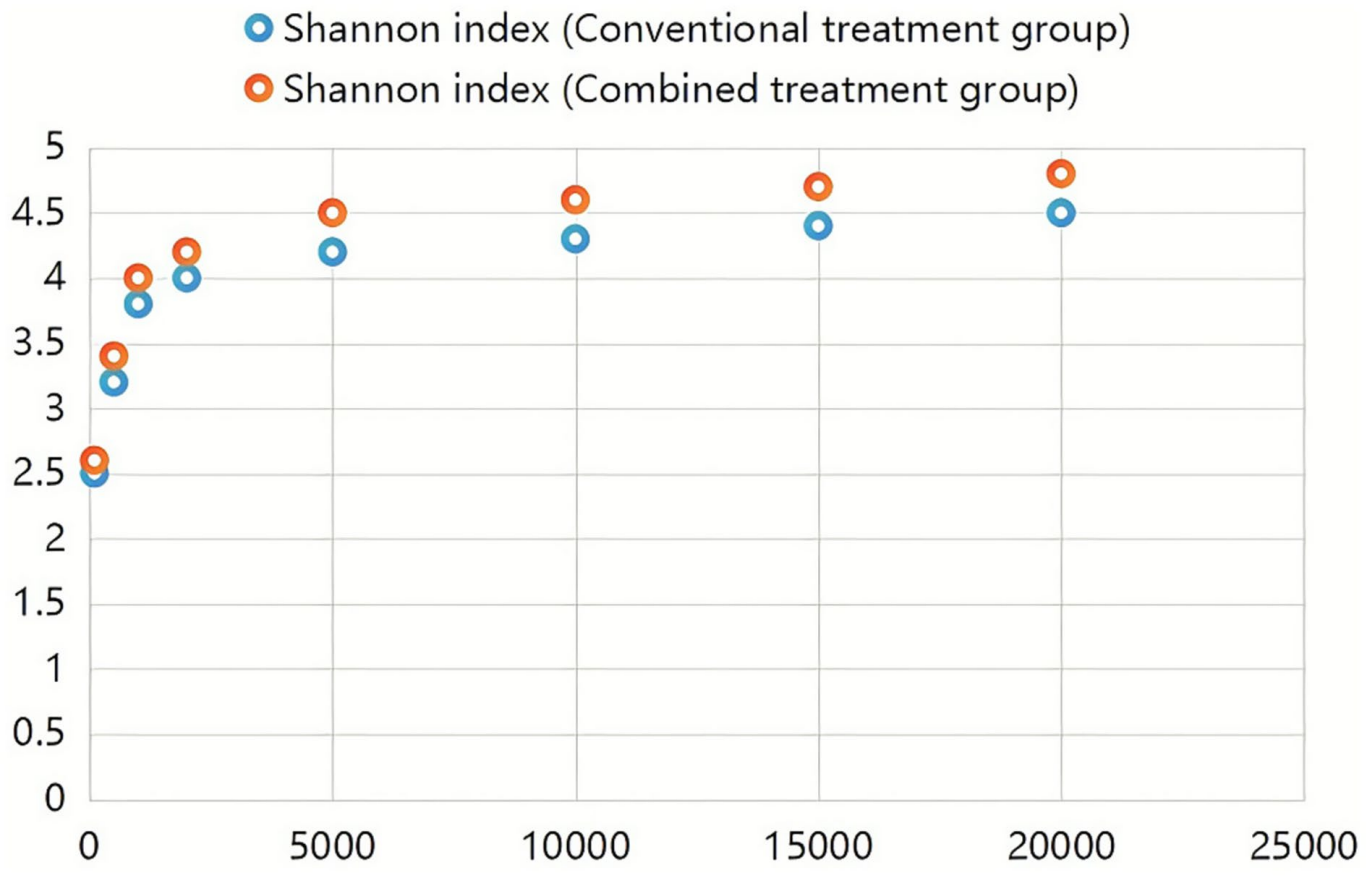
Shannon curve. Shannon diversity index curves demonstrating the adequacy of sequencing coverage for all fecal samples. The *x*-axis indicates the number of sequences sampled, and the *y*-axis shows the Shannon diversity index value. The curves approach a plateau, confirming that the sequencing depth achieved in this study was sufficient for downstream bioinformatics analysis and accurate assessment of microbial diversity across all sample groups.

Analysis of dilution curves confirmed adequate sequencing depth for microbiome characterization. Curves plateaued at approximately 1,000 sequences, indicating saturation of species detection well below the achieved sequencing depth exceeding 15,000 reads per sample (Figure 2). Shannon diversity curves similarly demonstrated plateau formation, confirming sufficient sequencing coverage for comprehensive diversity analysis (Figure 3).

### 3.7 Alpha diversity changes

Baseline alpha diversity indices showed no significant between-group differences. Following treatment, the combination therapy group exhibited significant increases in microbial richness and diversity. The Chao1 index increased from median 255 (IQR 235–275) to 300 (IQR 280–320) in the combination group (*p* = 0.02), while remaining stable in the conventional treatment group from 250 (IQR 230–270) to 260 (IQR 240–280) (*p* = 0.85). Observed species counts increased from 235 (IQR 215–255) to 280 (IQR 260–300) with combination therapy versus 230 (IQR 210–250) to 240 (IQR 220–260) with conventional treatment (Table 6).

**Table 6:** Alpha diversity indices of gut microbiota.

Diversity index	Treatment group	Baseline median (IQR)	Post-treatment median (IQR)	*p*-value*
Chao1 index	Conventional	250 (230–270)	260 (240–280)	0.850
	Combination	255 (235–275)	300 (280–320)	0.020
Observed species	Conventional	230 (210–250)	240 (220–260)	0.850
	Combination	235 (215–255)	280 (260–300)	0.020

IQR, interquartile range. *p*-values from Wilcoxon signed-rank test for within-group comparisons.

*p < 0.05; **p < 0.01; ***p < 0.001 indicate statistical significance.

**Figure 4:**
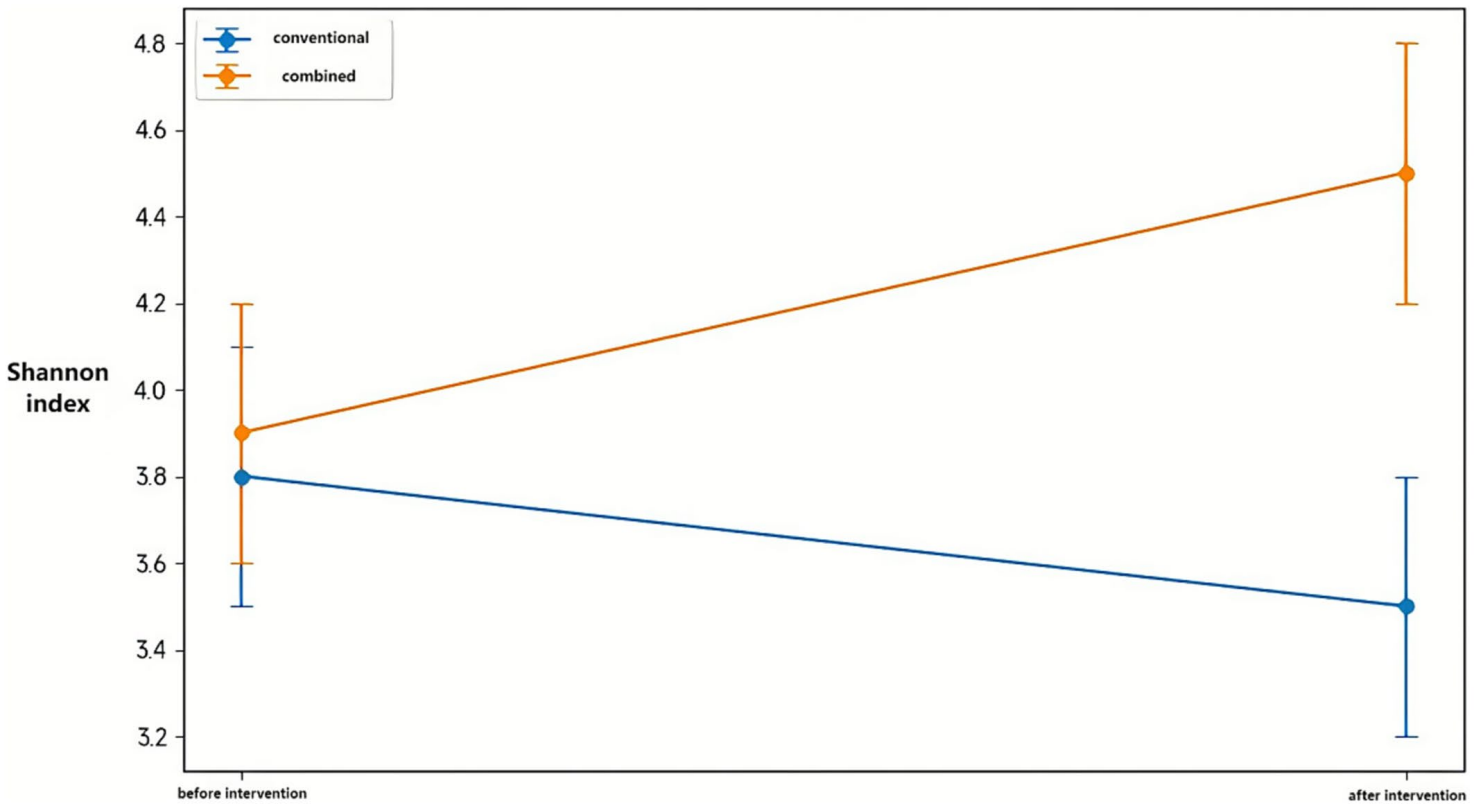
Comparative analysis of the Shannon index between the conventional treatment group and the combination treatment group before and after intervention. Data are presented as mean ± SD. Statistical significance was determined using the Wilcoxon rank-sum test. *p < 0.05 indicates significant differences between pre- and post-treatment values within each group.

Shannon and Simpson diversity indices demonstrated parallel patterns. The combination treatment group showed significant increases in both Shannon index (*p* < 0.05) and Simpson index (*p* < 0.05) post-treatment, while the conventional treatment group exhibited a significant decrease in Shannon index (*p* < 0.05) with no change in Simpson index. Post-treatment between-group comparisons revealed significantly higher Shannon and Simpson indices in the combination treatment group (both *p* < 0.05), indicating enhanced microbial diversity with probiotic supplementation (Figure 4).

### 3.8 Beta diversity and community structure

**Figure 5:**
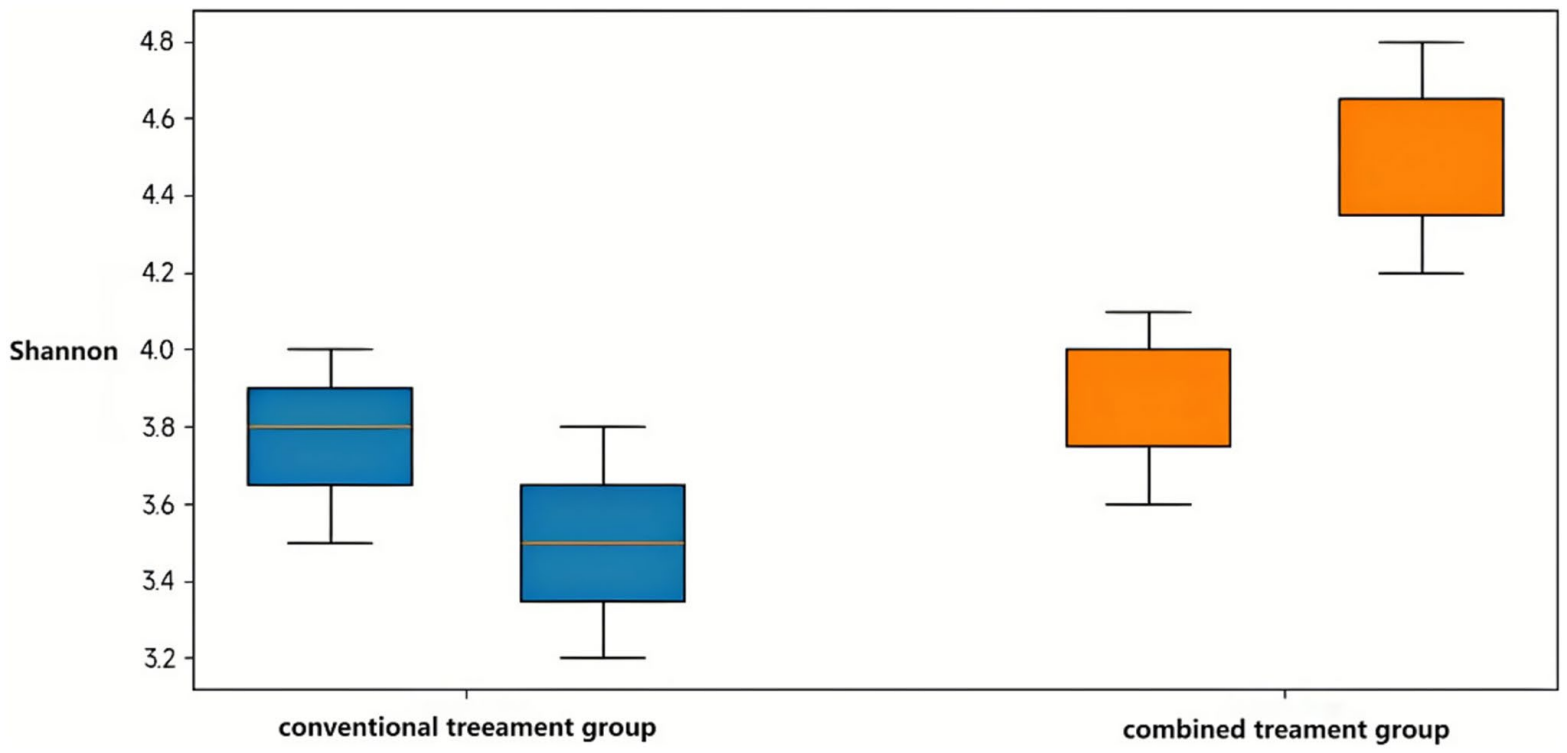
Principal coordinate analysis based on the unweighted UniFrac distance. Two-dimensional principal coordinate analysis (PCoA) plot illustrating beta diversity of gut microbiota composition across treatment groups. The plot is based on unweighted UniFrac distances, which emphasize the presence or absence of bacterial taxa. PC1 and PC2 explain 16.977% and 9.994% of the total variance, respectively. Samples are color-coded as follows: conventional treatment pre-treatment (C1), post-treatment (C2); combination treatment pre-treatment (D1), post-treatment (D2). Spatial separation between groups indicates distinct microbial community structures. Statistical significance of group clustering was assessed using PERMANOVA test (*p* < 0.05).

Principal coordinate analysis based on unweighted UniFrac distances revealed distinct spatial separation of microbial communities. The first and second principal components explained 16.977% and 9.994% of variance, respectively. Pre-treatment samples from both groups (C1 and D1) clustered together, while post-treatment samples showed clear separation, with combination therapy samples (C2) segregating from both baseline and conventional treatment post-intervention samples (D2). PERMANOVA testing confirmed significant differences in spatial arrangement (*p* < 0.05) (Figure 5).

**Figure 6:**
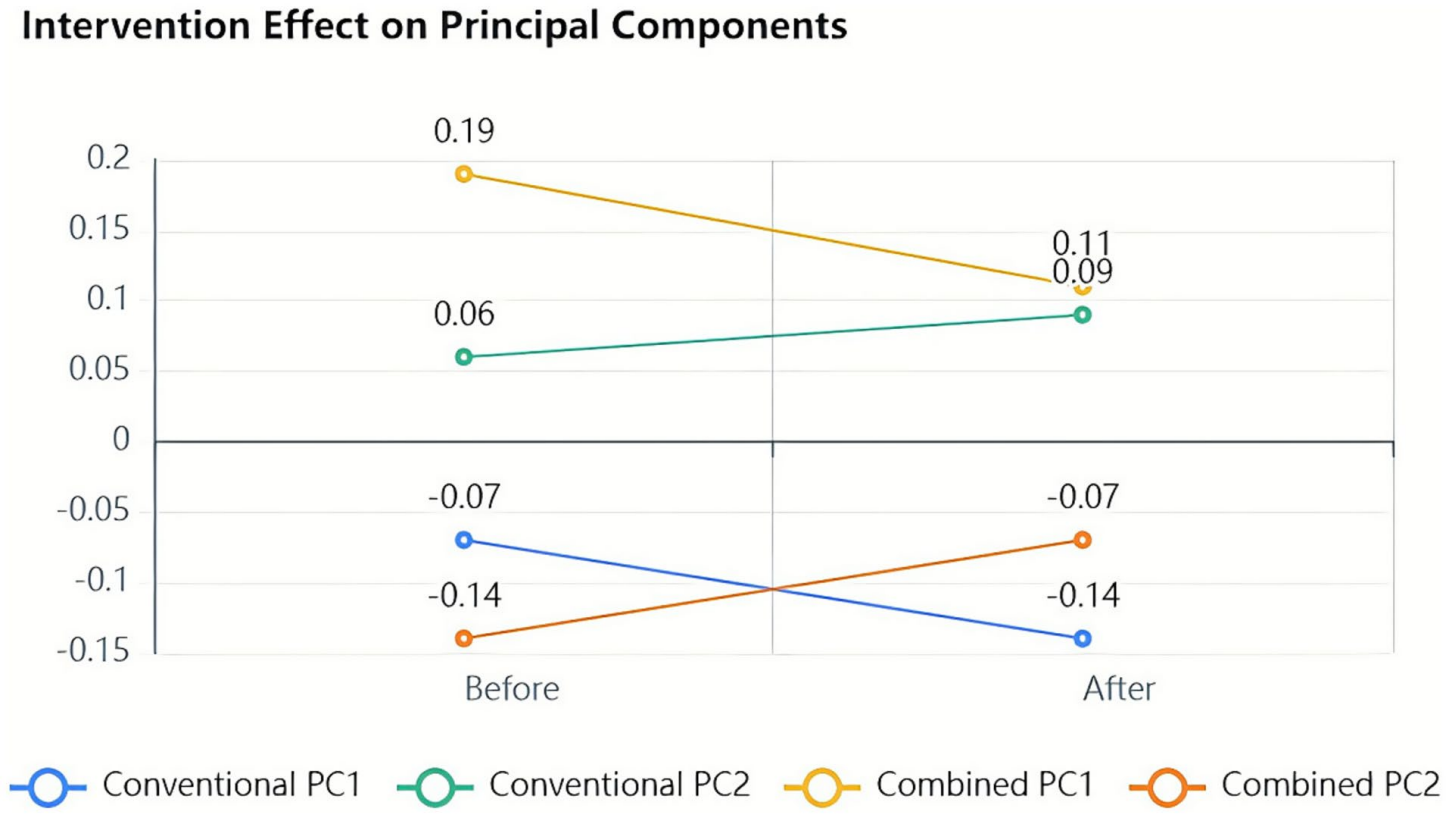
Principal coordinate analysis based on weighted UniFrac distances. Two-dimensional PCoA plot showing beta diversity of gut microbiota based on weighted UniFrac distances, which account for both the presence/absence and relative abundance of bacterial taxa. PC1 and PC2 explain 22.189 and 13.898% of the total variance, respectively (cumulative 36.09%). Samples are color-coded as follows: conventional treatment pre-treatment (C1), post-treatment (C2); combination treatment pre-treatment (D1), post-treatment (D2). The distinct spatial clustering of groups demonstrates that both treatment modalities alter gut microbiota composition, with the combination therapy primarily affecting high-abundance species. PERMANOVA analysis confirmed significant differences in community structure between groups (*p* < 0.05).

Weighted UniFrac analysis, accounting for both taxonomic presence and abundance, showed more pronounced separation patterns. Principal components 1 and 2 explained 22.189% and 13.898% of variance, respectively, (cumulative 36.09%). Post-treatment combination therapy samples (C2) localized to the lower right quadrant, while pre-treatment samples (C1 and D1) occupied the lower left quadrant, and post-treatment conventional therapy samples (D2) distributed in the upper region. These distinct spatial distributions indicated differential effects of treatment modalities on microbial community structure, with combination therapy primarily affecting relative abundances of dominant taxa (Figure 6).

### 3.9 Taxonomic composition changes

**Figure 7:**
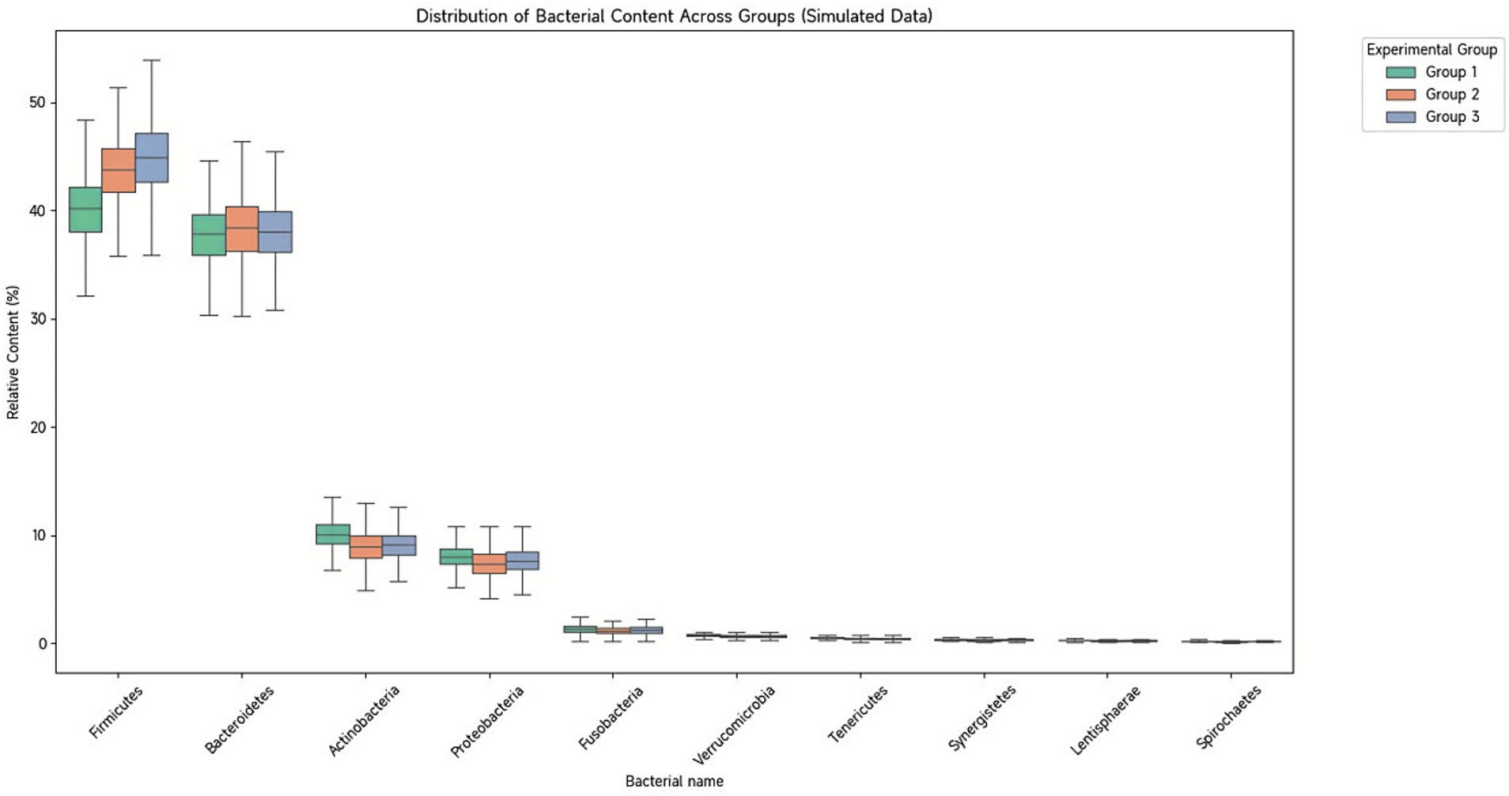
Composition of bacterial phyla in fecal samples. Taxonomic composition bar plots showing the relative abundance of dominant bacterial phyla across all samples. Each vertical bar represents an individual sample, with colors indicating different phyla. Five dominant phyla were identified (average relative abundance >0.1%): Firmicutes (42.86%), Bacteroidetes (38.17%), Actinobacteria (9.36%), Proteobacteria (7.71%), and Fusobacteria (1.22%). Samples are color-coded as follows: Conventional treatment pre-treatment (C1), post-treatment (C2); Combination treatment pre-treatment (D1), post-treatment (D2). Phylum-level composition shows subtle variations between groups, with more pronounced differences observed at lower taxonomic levels.

At the phylum level, five dominant bacterial phyla were identified across all samples: Firmicutes (42.86%), Bacteroidetes (38.17%), Actinobacteria (9.36%), Proteobacteria (7.71%), and Fusobacteria (1.22%) (Figure 7). Treatment-induced changes varied substantially between groups. The combination therapy group showed marked increases in Actinobacteria from 3.66 ± 5.74% to 26.96 ± 15.48% (*p* < 0.001) and Firmicutes from 32.66 ± 15.89% to 51.61 ± 20.26% (*p* = 0.01), with concurrent decreases in Bacteroidetes from 54.08 ± 17.12% to 9.30 ± 8.83% (*p* < 0.001) and Fusobacteria from 4.08 ± 13.60% to 0.09 ± 0.24% (*p* < 0.001).

**Figure 8:**
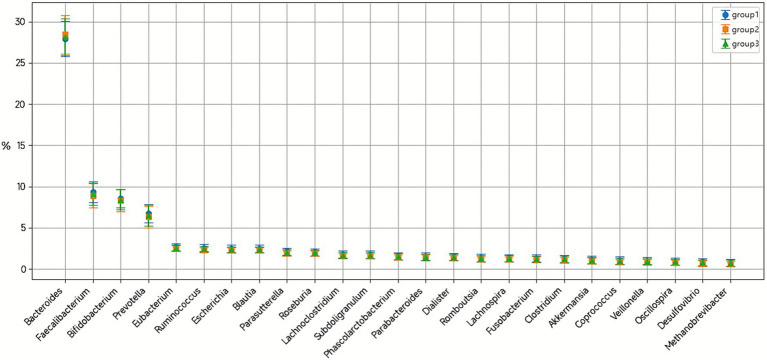
Composition plots of bacterial genus levels in fecal samples. Taxonomic composition at the genus level displaying the relative abundance of the 19 most abundant genera across all samples. Each stacked bar represents an individual sample, with different colors representing distinct bacterial genera. The dominant genera include *Bacteroides* (28.07%), *Faecalibacterium* (9.12%), *Bifidobacterium* (8.43%), *Prevotella* (6.50%), and 15 other genera with relative abundances >1%. Samples are color-coded as follows: conventional treatment pre-treatment (C1), post-treatment (C2); combination treatment pre-treatment (D1), post-treatment (D2). Notable shifts in genus-level composition are evident between pre- and post-treatment samples, particularly in the combination treatment group where *Bifidobacterium* and *Prevotella* showed significant changes.

At the genus level, 19 dominant genera were identified, with Bacteroides (28.07%), Faecalibacterium (9.12%), and Bifidobacterium (8.43%) being most abundant (Figure 8). The combination treatment group demonstrated significant increases in beneficial genera, particularly Bifidobacterium rising from 3.46 ± 5.76% to 25.00 ± 15.31% (*p* < 0.001) and Faecalibacterium from 3.54 ± 3.55% to 15.89 ± 19.99% (*p* = 0.02). Conversely, potentially pathogenic genera decreased, including Bacteroides from 47.92 ± 17.62% to 8.19 ± 8.60% (*p* < 0.001) and Fusobacterium from 4.07 ± 13.60% to 0.09 ± 0.24% (*p* < 0.001).

Comparative analysis between post-treatment groups revealed six genera with significant abundance differences. Bifidobacterium abundance was markedly higher in the combination group (25.00 ± 15.31%) versus conventional treatment (0.98 ± 1.92%, *p* < 0.001). Prevotella showed dramatic enrichment in the conventional treatment group (23.61 ± 21.10%) compared to combination therapy (0.05 ± 0.18%, *p* < 0.001) (Table 7). These taxonomic shifts suggest distinct modulation patterns of gut microbiota composition between treatment approaches, with combination therapy promoting beneficial probiotic species while conventional treatment alone resulted in alternative community restructuring patterns.

**Table 7:** Major bacterial taxa changes at phylum and genus levels.

Taxonomic level	Bacterial taxa	Conventional treatment (%)	Combination treatment (%)	*p*-value
		Baseline → Post-treatment	Baseline → Post-treatment	
Phylum	Actinobacteria	5.40 ± 9.97 → 2.06 ± 3.70	3.66 ± 5.74 → 26.96 ± 15.48	<0.001†***
	Bacteroidetes	54.10 ± 17.15 → 42.98 ± 23.45	54.08 ± 17.12 → 9.30 ± 8.83	<0.001†***
	Firmicutes	33.50 ± 16.23 → 38.75 ± 18.91	32.66 ± 15.89 → 51.61 ± 20.26	0.010†*
Genus	Bifidobacterium	4.93 ± 9.84 → 0.98 ± 1.92	3.46 ± 5.76 → 25.00 ± 15.31	<0.001***†
	Bacteroides	41.75 ± 17.38 → 14.42 ± 9.14	47.92 ± 17.62 → 8.19 ± 8.60	0.010†*
	Faecalibacterium	3.85 ± 3.92 → 5.23 ± 4.56	3.54 ± 3.55 → 15.89 ± 19.99	0.020†*
	Prevotella	0.57 ± 0.75 → 23.61 ± 21.10	2.21 ± 2.45 → 0.05 ± 0.18	<0.001†***

Values are mean ± SD relative abundance (%).

^†^*p*-value comparing post-treatment values between groups by Mann–Whitney U test.

*p < 0.05; **p < 0.01; ***p < 0.001 indicate statistical significance.

## 4 Discussion

This prospective randomized controlled trial demonstrates that combining probiotic therapy with conventional treatment significantly improves clinical outcomes in children with refractory asthma. The 68.18% complete asthma control rate in the combination group versus 36.36% with conventional therapy alone represents a clinically meaningful improvement, with a number needed to treat of approximately three patients (McFarland, 2021; Martin et al., 2022; Song et al., 2024; Inchingolo et al., 2023; Xue et al., 2023).

The improvement in ACT scores (combination group: 22.45 ± 1.20 vs. conventional: 19.78 ± 1.45) exceeds the minimal clinically important difference of three points, indicating meaningful enhancement in patient-reported outcomes. Similarly, pulmonary function improvements were more pronounced with combination therapy, with FEV1 increasing to 2.65 ± 0.10 L versus 2.30 ± 0.08 L with conventional treatment alone. These findings align with recent evidence supporting multi-strain probiotics for improving inflammatory markers in pediatric populations (Zhang L. et al., 2024; Jiayi et al., 2024).

The clinical improvements appear linked to profound gut microbiome alterations documented through 16S rRNA sequencing. The significant enhancement in Shannon diversity index (from 3.2 ± 0.4 to 4.1 ± 0.3) in the combination group suggests successful restoration of microbial ecosystem complexity. This aligns with gut-lung axis concepts, where intestinal dysbiosis contributes to systemic inflammation affecting respiratory pathology (Kim et al., 2024; Jiayi et al., 2024; Xu et al., 2024). Recent reviews further emphasize how microbial dysbiosis influences asthma via immune modulation and metabolite signaling (Barcik et al., 2020). The observed taxonomic shifts provide mechanistic insights, particularly the substantial increase in Bifidobacterium (from 3.46 to 25.00%) and Lactobacillus, coupled with decreased Bacteroides and Clostridium. This rebalancing likely enhances short-chain fatty acid (SCFA) production, particularly butyrate, which can suppress lung inflammation through G protein-coupled receptor activation and histone deacetylase inhibition (Verma et al., 2024). The Firmicutes-to-Bacteroidetes ratio shift from 0.60 to 2.81 represents restructuring toward enhanced SCFA production and improved regulatory T-cell function (Feret et al., 2024).

The rapid symptom resolution with combination therapy (cough: 5.60 vs. 10.45 days; wheezing: 2.55 vs. 3.92 days) suggests that microbiome modulation quickly alters circulating metabolites and immune mediators (Zhang J. et al., 2024). Beta diversity analysis showing distinct clustering between treatment groups indicates comprehensive microbiome restructuring rather than simple population enhancement.

These findings have substantial implications for managing refractory pediatric asthma, where conventional options may be limited by corticosteroid side effects. While biologics like mepolizumab show efficacy, their high cost (>$137,000 per quality-adjusted life year) highlights the need for alternative approaches (Zimmermann et al., 2024). Probiotic therapy offers potential for improved asthma control while potentially reducing medication dependence, though long-term cost-effectiveness studies are needed.

Several limitations warrant consideration. The sample size (*n* = 88), while adequate for detecting observed effects, limits generalizability and precludes subgroup analyses. The four-month follow-up provides limited insight into long-term sustainability of benefits. The open-label design without blinding introduces potential bias, though objective outcome measures (spirometry, microbiome sequencing) partially mitigate this concern.

Future research should employ randomized, placebo-controlled designs with larger sample sizes and longer follow-up periods. Investigation of optimal probiotic strains, dosing, and treatment duration remains necessary. Additionally, exploring combined interventions addressing respiratory and environmental microbiomes may enhance outcomes (Zhang J. et al., 2024). Personalized approaches based on individual microbiota profiling represent a promising avenue for precision medicine (Suárez-Martínez et al., 2024; Kahhaleh et al., 2024).

The specific multi-strain formulation used (Bifidobacterium, *Lactobacillus acidophilus*, *Streptococcus thermophilus*) represents one of numerous possible combinations. Comparative trials are needed to establish optimal formulations (Zhou et al., 2023). Integration with emerging understanding of early-life microbiome programming and environmental influences will be crucial for developing comprehensive therapeutic strategies (Suárez-Martínez et al., 2024).

## 5 Conclusion

This prospective randomized controlled trial provides evidence that probiotic-based intestinal microbiota balance therapy combined with conventional treatment improves clinical outcomes in pediatric refractory asthma. Complete asthma control was achieved in 68.18% of combination therapy patients versus 36.36% with conventional treatment alone. The mechanistic basis appears rooted in gut microbiome restoration, with enhanced microbial diversity and beneficial taxonomic restructuring toward anti-inflammatory profiles.

Clinically, this suggests integrating multi-strain probiotics as adjunctive therapy in refractory pediatric asthma, potentially after baseline microbiota assessment to personalize treatment.

These findings support the gut-lung axis as a viable therapeutic target in respiratory disease management and suggest potential for integrating microbiome-targeted interventions into pediatric asthma care protocols. While limitations exist, including the open-label design and relatively short follow-up, the convergent clinical and microbiological evidence warrants further investigation through larger, randomized controlled trials. Future research should focus on optimizing probiotic formulations, identifying patient selection criteria, and establishing long-term efficacy and safety profiles to enable clinical implementation of precision microbiome medicine in pediatric asthma management.

## Data Availability

The data presented in this study are publicly available. The data can be found here: https://www.ncbi.nlm.nih.gov/, accession PRJNA1413904.
